# Chitosan-Based Green Pea (*Pisum sativum* L.) Pod Extract Gel Film: Characterization and Application in Food Packaging

**DOI:** 10.3390/gels9020077

**Published:** 2023-01-18

**Authors:** Essam Mohamed Elsebaie, Mona Metwally Mousa, Samah Amin Abulmeaty, Heba Ali Yousef Shaat, Soher Abd-Elfttah Elmeslamy, Galila Ali Asker, Asmaa Antar Faramawy, Hala Ali Yousef Shaat, Wesam Mohammed Abd Elrahman, Hanan Salah Eldeen Eldamaty, Amira Lotfy Abd Allah, Mohamed Reda Badr

**Affiliations:** 1 Food Technology Department, Faculty of Agriculture, Kafrelsheikh University, Kafr El-Sheikh 33516, Egypt; 2 Food Science &Technology Department, Faculty of Home Economics, Al-Azhar University, Tanta 31732, Egypt; 3 Nutrition & Food Science Department, Faculty of Home Economics, Al-Azhar University, Tanta 31512, Egypt; 4 Food Science and Technology Department, Agriculture Faculty, Tanta University, Tanta 31512, Egypt

**Keywords:** green pea pods, corn oil, chitosan, films, gel

## Abstract

This work focuses on studying the preparation, characterization (physical, mechanical, optical, and morphological properties as well as antioxidant and antimicrobial activities) and packaging application of chitosan (CH)-based gel films containing varying empty green pea pod extract (EPPE) concentrations (0, 1, 3, and 5% *w*/*w*). The experiments revealed that adding EPPE to CH increased the thickness (from 0.132 ± 0.08 to 0.216 ± 0.08 mm), density (from 1.13 ± 0.02 to 1.94 ± 0.02 g/cm^3^), and opacity (from 0.71 ± 0.02 to 1.23 ± 0.04), while decreasing the water vapour permeability, water solubility, oil absorption ratio, and whiteness index from 2.34 to 1.08 × 10^−10^ g^−1^ s^−1^ pa^−1^, from 29.40 ± 1.23 to 18.75 ± 1.94%, from 0.31 ± 0.006 to 0.08 ± 0.001%, and from 88.10 ± 0.43 to 77.53 ± 0.48, respectively. The EPPE films had better tensile strength (maximum of 26.87 ± 1.38 MPa), elongation percentage (maximum of 58.64 ± 3.00%), biodegradability (maximum of 48.61% after 3 weeks), and migration percentages than the pure CH-gel film. With the addition of EPPE, the antioxidant and antibacterial activity of the film improved. SEM revealed that as EPPE concentration increased, agglomerates formed within the films. Moreover, compared to control samples, packing corn oil in CH-based EPPE gel films slowed the rise of thiobarbituric acid and peroxide values. As an industrial application, CH-based EPPE films have the potential to be beneficial in food packaging.

## 1. Introduction

Packaging is a crucial part of the food industry because it helps with food handling, storage, transportation, and preservation, as well as protecting against external contaminants, preventing substances inside the food from escaping into the surrounding environment, and reducing food waste [1,2]. Plastic polymers are commonly used for packaging because they are easy to manufacture, inexpensive, printable, and highly resistant to various mechanical and environmental variables [3]. Nevertheless, these packaging substances are harmful to the environment since they take a long time to disintegrate and present the danger of releasing chemicals that might affect food quality [4].

Therefore, the use of biodegradable packaging rather than plastic packaging is encouraged due to environmental and health concerns. Nowadays, the basic substance in biodegradable packaging films is usually derived from natural bio-polymers, such as polysaccharides, lipids, and proteins [5]. These may be recycled, degrade quickly, are non-toxic, and are environment-friendly [6,7].

Chitosan (CH) is the deacetylated chitin derivative and the second most frequent polysaccharide found naturally after cellulose. It is a linear polysaccharide composed of (1,4)-linked 2-amino-deoxy-β-D-glucan [8]. CH has demonstrated benefits in the creation of biodegradable films because of its unique characteristics, including ease of film formation, nontoxicity, good mechanical strength, excellent barrier capacity, antioxidant activity, biodegradability, and antimicrobial activity [9]. Despite the benefits mentioned above, a pure CH film is frequently fragile, has low force resistance, and is relatively susceptible to moisture [10]. Furthermore, continued development of its antioxidant and antibacterial characteristics is required for active packaging, and therefore, that becomes the focus of attention over time. Many modification procedures have been presented so far, such as the addition of antioxidants, antibacterial compounds, and reinforcing agents to either transmit or enhance certain characteristics of CH-based films [11]. The use of antioxidants in packaging films has grown in popularity since oxidation is a key issue impacting the quality of food. Butylated hydroxyl-toluene (BHT) and butylated hydroxy-anisole (BHA) are the most commonly added antioxidants to active packaging films nowadays [12]. Even though the great stability, low cost, and efficiency of these artificial antioxidants make them an effective choice for active food packaging, there are serious concerns about their toxicological properties [13].

Furthermore, due to the possible health risks posed by these substances, the consumption of artificial antioxidants is strictly regulated. In order to replace artificial antioxidants with natural ones, such as polyphenolic compounds, extensive investigations have been done in this area [14,15]. Several plant extracts have been incorporated into the CH film as a source of antioxidants, such as murta leaf extract [16], sweet potato extract [17], grape seed extract [18], banana peels extract [19], soybean seed coat extract [20], pomegranate peel extract [21], chestnut extract [22], etc., resulting in a boost in the film’s antioxidant properties.

Pea *(Pisum sativum)*, sometimes referred to as “Besela” in Egypt, is an annual crop grown during the winter [23]. It is one of Egypt’s most significant vegetable crops, and cooked green seeds are one of the most popular foods consumed. Empty pea pods (EPP), which make up between 30 and 67% of the entire weight of the pod, are a by-product of the pea processing sector [24]. Several high-value compounds are abundant in these EPP. They include a high amount of proteins, dietary fibre (over 50%, mostly water-insoluble), iron, potassium, and phenolic components [25]. The latter has significant promise and may be used in the feed, food, pharmaceutical, and cosmetic sectors if it is made from a cheap resource such as EPP [26]. Although the chemical composition of EPP has been extensively studied, little is currently known about its polyphenol compounds. Catechin, epicatechin, gallic acid, and other phenolic components found in abundance in empty pea pod extract (EPPE) provide EPP with a high antioxidant capacity [25,26].

To the best of our knowledge, no studies have been performed on the use of EPP or EPPE as a possible natural addition to boost the antioxidant activity of biodegradable film. Scientific research with the aim of developing active food packaging sheets incorporating EPPE might provide the pea processing sector with a new functionality and turn EPP into a very precious resource. Thus, the current study intended to develop an innovative CH-based active packaging gel film for the first time by incorporating EPPE and to assess the physical, optical, mechanical, morphological, and biological characteristics of the CH films produced. The gel films produced were also assessed as a package for oil storage because of their potential use in slowing the oxidation of corn oil.

## 2. Results and Discussion

### 2.1. Physical Properties

#### 2.1.1. Film Thickness and Density

The thickness and density of a formulation are signs of its reliability and the quality preparation process. The film thickness and density values rose as the concentration of EPPE in the gel films increased, as indicated in Table 1. The film density and thickness both changed significantly (*p* < 0.05). Chitosan gel films that had been supplemented with 5% EPPE (Ch-5% EPPE) had the maximum film thickness (0.216 ± 0.08 mm) and density (1.94 ± 0.02 g cm^−3^), whereas control films had the lowest values of thickness (0.132 ± 0.08 mm) and density (1.13 ± 0.02 g cm^−3^) (Table 1). Riaz et al. [27] found a similar result, reporting that increasing the amount of apple peel polyphenolic extract in the chitosan gel matrix enhances film thickness and density. Peng et al. [28] discovered that with an increased amount of extract added to the film, the interaction between polyphenol components and CH increases. This increasing interaction causes stronger composite binding as the space between both the interacting molecules becomes smaller, enhancing film thickness and density with a rise in the applied EPPE’s concentration in the CH-gel matrix.

#### 2.1.2. Film Water Vapour Permeability (WVP)

A film’s WVP coefficient is a constant value for water vapour permeability at a specific temperature. A film’s permeability is influenced by its chemical composition, morphology, type of permanence, and ambient temperature. WVP is a metric that measures how much moisture may pass through a film. It is more crucial in food preservation to protect a substance from moisture. The results of an investigation into the water vapour permeability of CH-gel films containing various EPPE concentrations are shown in Table 1. The results revealed that raising the EPPE concentration in the CH-gel matrix lowered the film’s WVP. Significant changes (*p* < 0.05) were seen between all prepared films. The control film (Ch-0% EPPE) had the maximum WVP (2.34 ± 0.14 × 10^−10^ g^−1^ s^−1^ pa^−^**^1^**), followed by Ch-1% EPPE (1.58 ± 0.09 × 10^−10^ g^−1^ s^−1^ pa^−^**^1^**), Ch-3% EPPE (1.32 ± 0.07 × 10^−10^ g^−1^ s^−1^ pa^−^**^1^**), and Ch-5% EPPE (1.08 ± 0.06 × 10^−10^ g^−1^ s^−1^ pa^−^**^1^**). The lower WVP might be attributed to the restricted interaction between water molecules in the film as a result of the cross-linking action of chitosan, glycerol, and EPPE, culminating in less available free water [29]. The low WVP value for food packaging sheets is extremely desirable to reduce moisture transfer between the food and its immediate environment. The films’ thickness is an important factor in defining their water barrier characteristics. WVP values are lower in thicker films, as water molecules take longer to flow through the film. Kurek et al. [30] observed comparable behaviour after incorporating blueberry and blackberry extracts into chitosan-based gel films.

#### 2.1.3. Film Water Solubility (WS)

Greater solubility in water can increase film biodegradability by decreasing its degradation period in the environment. Furthermore, it will lessen the application of film on foods with high water content [29]. An increase in EPPE’s addition percentage resulted in a decrease in EPPE films’ WS values. Ch-1% EPPE film did not vary significantly (*p* < 0.05), while Ch-3% EPPE and Ch-5% EPPE films demonstrated a substantial reduction (*p* < 0.05) in WS in comparison to the control (Ch-0% EPPE). The WS of the EPPE films decreased, as demonstrated in Table 1, as a result of the extract’s polyphenolic components forming strong hydrogen bonds with the CH matrix. Both Uranga et al. [31] and Riaz et al. [32] observed similar findings.

#### 2.1.4. Film Oil Resistance Ability

The oil absorption ratio was used to assess Ch-EPPE gel films’ capacity for oil resistance in possible food packaging use (OAR). The findings revealed that the Ch-5% EPPE film had a lower OAR percentage (0.08 ± 0.001%) than the control film (Ch-0%EPPE), which had an OAR percentage of 0.31 ± 0.006% (Table 1). Ch-1% EPPE, Ch-3% EPPE, and Ch-5% EPPE films all showed a substantial reduction in OAR percentages, from 0.26 ± 0.004 to 0.08 ± 0.001%. This reduction may be due to the fact that CH contains a hydrophilic hydroxyl group in its structure, which acts with EPPE to increase the film thickness, making it more difficult for oil molecules to cross through them. As a result, low OAR values demonstrated greater oil resistance capacity, which is a favourable attribute for packaging materials for oil goods. Riaz et al. [32] observed similar findings.

### 2.2. Film Colour and Opacity

The nutritional and flavour protection of food once exposed to visible and ultraviolet light are both determined by the optical properties of food packaging films [33,34]. These properties also impact how well the film is tolerated by a user. Colour parameters, whiteness index (WI), and opacity measurements were used to assess the prepared gel films’ optical properties. Compared with the control gel film (Ch-0% EPPE), Ch-EPPE films showed significantly (*p* < 0.05) lower levels of L*. This demonstrated that the darkness intensity of Ch-EPPE gel films increases as their EPPE content increases. The films were darker as a result of light scattering and refraction produced by phenolic EPPE components [35]. Ch-5% EPPE film showed a maximum 11.94% reduction in lightness compared to the control film (Ch-0% EPPE).

Chitosan gel films’ b* (yellowness/blueness) and a (redness/greenness) values were considerably (*p* < 0.05) impacted by the addition of EPPE. As the EPPE concentrations climbed from 0 to 5%, the gel films’ b* values increased from (1.03 ± 0.01) to (1.98 ± 0.04) (an indication of the trend towards yellowness) and their a* values from 0.84 ± 0.01 to 1.12 ± 0.03 (an indication of the trend towards redness).

Based on these results, the existence of phenolic components and coloured compounds inside the integrated EPPE as well as the inner structure formed during film drying might be responsible for the addition of EPPE’s remarkable effect on the colour of the resultant Ch-EPPE gel films [35,36]. Similar colour measurement findings were achieved when green tea extract and CH were combined, producing films that were a deeper shade of greenish yellow [29]. The films’ level of whiteness and opacity were determined using opacity tests and a WI estimate. The WI of the Ch-EPPE was substantially different (*p* < 0.05) from that of the pure CH- film, as indicated in Table 2.

The reduced WI for Ch-EPPE films suggested that the film was beginning to become darker, allowing for the preservation of light-sensitive food items [36]. Opacity measurements revealed that the Ch-EPPE films were significantly (*p* < 0.05) impenetrable, with the Ch-5% EPPE film having a 73.24% increase in opacity over the control (Ch-0% EPPE). Because the chitosan polymer backbone has a mostly linear structure and offers the least resistance to light penetration compared to globular plasticizers, a comparison of the WI and opacity values of all the Ch-EPPE films with the control reveals a small quantitative drop in whiteness and a small rise in opacity [37]. Siripatrawan and Harte [29] obtained similar results.

### 2.3. Mechanical Properties

A film’s greatest ability to endure applied tensile stress is measured by its tensile strength (TS), while its capacity to stretch is shown by its elongation percentage (EB%) [38]. Films with good mechanical characteristics are advantageous in industrial manufacturing, packing, transporting, and end-use applications. A comparative statistical analysis was done on the mechanical characteristics of chitosan gel films that contained various EPPE concentrations (Table 3). When the EPPE percentage rose from 0 to 5%, the TS and EB% both improved considerably, from 21.30 ± 1.191 to 26.87 ± 1.38 MPa and from 53.42 ± 3.02 to 58.64 ± 3.00%, respectively. The interaction between both the CH matrix and the polyphenolic components from EPPE may be responsible for the impact of EPPE inclusion on the enhancement in the mechanical characteristics of the related films. These interactions may result in tighter polymer chain-to-chain connections and better interfacial bonding between the CH monomers and the EPPE in the gel film layer, both of which increase the resistance against mechanical stress [39]. Similar findings were made by Balti et al. [40] and Siripatrawan and Harte [29], who noted that TS and EB increased as *spirulina* extract or green tea content increased from 0 to 5%.

### 2.4. Bioactivities of CH-EPPE Film

#### 2.4.1. Biodegradation Evaluation

**Figure 1:**
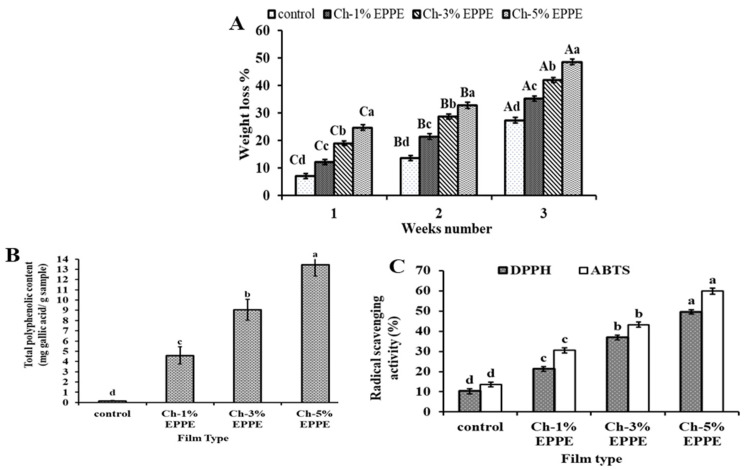
Biodegradability evaluation of chitosan films incorporated with EPPE (**A**), total phenolic content (**B**), and scavenging activities of the EPPE films on DPPH and ABTS radicals (**C**). Different letters indicate significant differences (*p* < 0.05). Values are given as mean (*n* = 3) ± SD.

The high biodegradability of a film by reducing its time of degradation in the environment is an important property that the new packaging must have because it reduces environmental problems [28]. The biodegradation of CH and Ch-EPPE gel films is depicted in Figure 1A. The weight loss of the Ch-EPPE and control (ch-0% EPPE) gel films increased (*p* < 0.05). As the duration for ground dumping was extended to 3 weeks, the weight loss rapidly accelerated for all the films examined. After three weeks, the Ch-5% EPPE film had the maximum weight reduction of 48.61%, compared to 27.35% for the control film (Ch-0%EPPE). A similar tendency towards biodegradation was observed when Chinese chive root extract was added to the CH-based sheet [32]. The main finding of the biodegradation assessment in this study was that adding EPPE to CH films enhance biodegradability by creating new polymer composites and reducing the environmental load through rapid breakdown.

#### 2.4.2. Antioxidant Properties

Total phenolic content (TPC) and two other types of tests were used to assess the synthetic films’ antioxidant activity (DPPH and ABTS). Figure 1 displays the results of the antioxidant activity. For active packaging sheets, TPC was utilized as a preliminary antioxidant evaluation [41]. Due to their capacity for free electron delocalization and H+ ion (of the hydroxyl group) donation, phenolic substances have been shown to have antioxidant action [42]. Figure 1B displays the TPC of CH edible films combined with EPPE. The findings revealed that as EPPE concentration increased, the total phenolic content of the CH films increased significantly (*p* < 0.05) (Figure 1B). In addition, CH films that did not have EPPE had a low TPC of 0.18 ± 0.003 mg GAE/g film. This finding may be explained by the generation of chromogens as a result of the Folin and Ciocalteu reagents reacting with non-phenolic reducing agents that may be identified spectrophotometrically [43]. Along with its phenolic components, EPPE’s antioxidant action may also be attributed to other possible constituents, including the flavonoids that are present [25]. The DPPH and ABTS tests are therefore critically important in evaluating these components’ antioxidant activity. As can be seen in Figure 1C, the CH- EPPE films’ %DPPH radical scavenging was significantly (*p* < 0.05) greater than that of the pure CH films. The DPPH radical scavenging of the Ch-5% EPPE film was 49.71%, which was 4.83 times higher than that of the control (Ch-0% EPPE) film. The conclusions of the DPPH investigation followed a trend similar to the ABTS radical scavenging findings (Figure 1D). Films containing EPPE revealed significantly higher ABTS scavenging activity than the control (*p* < 0.05). The highest ABTS cation elimination (59.89%) was seen in the Ch-5% EPPE film as a result of its improved availability of antioxidant content. This increment was 4.40 times greater than the control group. When the antioxidant evaluations for the Ch-EPPE films were compared to one another, each antioxidant assay indicated a significant (*p* < 0.05) rise. This demonstrated how little the CH polysaccharide chain contributed to the antioxidant action [44].

#### 2.4.3. Migration Test

Active packaging emits the active component via migration through the headspace or direct contact with the food’s surface. Hence, it is crucial to assess the active packaging’s capacity for releasing the active component using a migration experiment. For the migration experiment, the Food and Drug Administration of the United States has advised using food mimics, such as water (to simulate an aqueous medium) and 95% ethanol (to represent a fatty medium) [45]. The polyphenol data revealed that there is a movement of polyphenolic compounds that is greater in ethanol than in water (Table 4). Kurek et al. [46] maintain that if the structural integrity of the film is preserved, the active ingredient will continue to be present. This also has an impact on the increased GAE observed in ethanol compared with water. The Ch-5% EPPE film has the greatest migration value (5.09 ± 0.06 mg gallic acid/mL water and 9.12 ± 0.08 mg gallic acid/mL ethanol), which differs substantially (*p* < 0.05) from all other measures of polyphenol migration. It is preferable for active ingredients to migrate, such as polyphenols and antioxidants, since they can enhance the qualities of packed food items, prevent oxidation, increase their shelf life, and serve as a package having active characteristics [47].

#### 2.4.4. Antimicrobial Activity

For the food to be shielded from microbial development and kept fresh for a long time, the active food packaging sheet must have strong antimicrobial activity [48]. Table 5 displays the antibacterial activity of four Gram-positive and Gram-negative bacteria against CH edible gel films combined with EPPE at various percentages.

As shown in Table 5, the control films (Ch-0%-EPPE) were ineffective against either of the four bacterial strains, but with the addition of EPPE, all the tested films showed antimicrobial activity on the contact area beneath the film discs. Our results were in accordance with those of Wang et al. [49], who found no significant inhibitory zone for the pure CH-gel film towards both Gram-positive and Gram-negative bacteria. This impact of CH may be connected to the fact that in the agar diffusion test method, chitosan does not diffuse across the neighbouring agar medium, meaning that only bacteria in direct contact with the active discs of CH are inhibited. The positively charged amino groups of the CH molecules, which may interact with the anionic groups on the microbial cell membrane, are also necessary for the antibacterial efficacy of chitosan [49]. In such instances, CH has been shown to exhibit intrinsic antibacterial activity against both Gram-positive and Gram-negative bacteria [22]. The Ch-EPPE films, in general, showed inhibitory effects (*p* < 0.05) on both Gram-positive and Gram-negative bacteria, and inhibition zones grew larger as the EPPE percentage rose in the film. According to the data reported in the same table, *Salmonella typhimurium* is the most sensitive to the films, followed by *E. coli, Pseudomonas aeruginosa,* and *Bacillus subtilis*. According to this study, EPPE worked better against Gram-positive bacteria than it did against Gram-negative bacteria. This could be due to variations in cell wall structure, as the cell walls of Gram-negative bacteria have lipopolysaccharides, which may prevent active constituents from entering the cytoplasmic membrane [50,51]. The primary location of contact for polyphenols with bacteria is the outer cell membrane [52]. In the polyphenols, the hydroxyl groups, conjugated double bonds, and galloyl groups are the active groups in charge of this interaction. The bacteria may die if the membrane, which protects the cell’s integrity, is damaged as a result of this contact.

### 2.5. Films SEM Photographs

**Figure 2:**
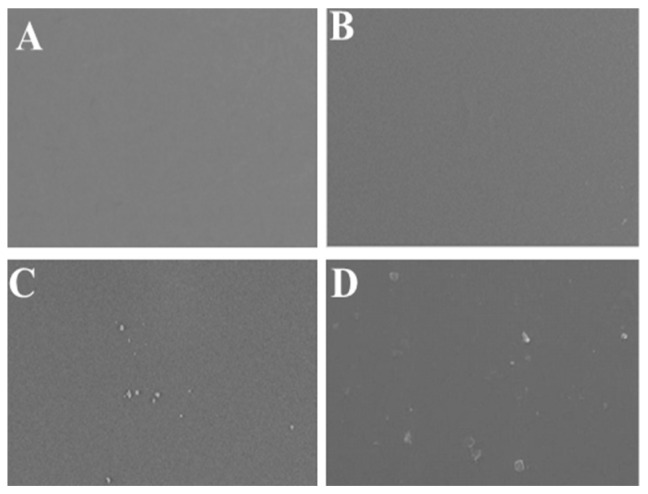
Active films’ SEM images, where (**A**) Control (Ch-0% EPPE), (**B**) Ch-1% EPPE, (**C**) Ch-3% EPPE, and (**D**) Ch-5%EPPE.

Figure 2A–D depict the results from a prepared film SEM study. The control gel film (ch-0%-EPPE) has a smooth, uniform surface. There were no impurities, delamination, or precipitates (Figure 2A). The studied films’ surface morphology was unaffected by the addition of 1% EPPE, it appears (Figure 2B). Small agglomerates were seen when CH was added with 3% EPPE (Figure 2C). A more heterogeneous surface was produced as a result of the structure being disturbed by the rise in EPPE concentration from 3% to 5%, which led to the appearance of more and more white patches (Figure 2D). This could be because EPPE contains hydrophilic polyphenolic components.

### 2.6. Application of Films in Packaging Edible Oil

**Figure 3:**
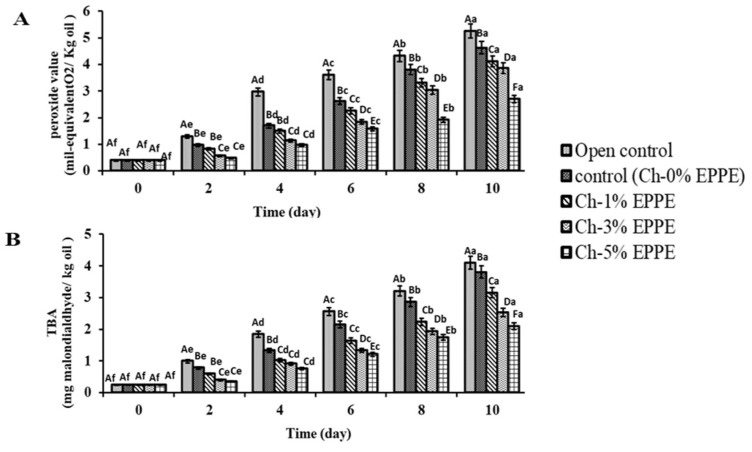
Changes in the PV (**A**) and TBARS (**B**) levels in corn oil stored in Ch-EPPE films at 50 °C for 10 days. Data are presented as means ± SD of triplicates. Different lowercase letters indicate the statistically significant difference (*p* < 0.05) within the same treatment group at different storage times. Different uppercase letters indicate the statistically significant difference (*p* < 0.05) among different treatment groups at the same storage time.

Edible oil oxidation occurs as a result of the effects of temperature, oxygen, and light [53]. In this context, oxidation stability is regarded as a crucial indicator of edible oil quality. Corn oil packed in four CH-based EPPE films (0, 1, 3, and 5%) and unpackaged corn oil (open control) were both evaluated for oxidation stability over a 10-day period at 50 °C. During oil storage, the values of peroxide value (PV) and thiobarbutic acid (TBA) steadily rose (Figure 3).

The oil’s PV and TBARS levels were significantly reduced in the CH-based EPPE films. All treatments produced higher PV and TBARS values independent of duration; however, this increase was slower for CH-based EPPE films than with open control and Ch-0% EPPE films. The open control had the greatest values of PV (5.26 ± 0.41 mil-equivalent O2/kg oil) and TBARS (4.13 ± 0.38 mg malondialdhyde/kg oil) on the 10th day of storage, whereas Ch-5% EPPE had the lowest values of PV (2.73 ± 0.24 mil-equivalent O2/kg oil) and TBA (2.10 ± 0.07 mg malondialdhyde/kg oil). The bio-composite film’s tight structure has an important role in reducing oxidation by making oxygen harder to get through [54]. Additionally, the inclusion of phenolic compounds in the film boosted the antioxidant potential of the sheet, which also aided in slowing the oxidation of the oil. Therefore, it may be assumed that CH-based EPPE films could be utilized as packaging films for foodstuffs that are extremely vulnerable to oxidation.

## 3. Conclusions

The empty pea pods resulting as residue from food factories are one of the sources that can be used in the separation of many important biological compounds, including phenolic compounds. The phenolic compounds present in EPPE have antioxidant and antimicrobial properties. In this study, active food films were prepared from chitosan-containing EPPE. The properties of these films were evaluated, and they were used to extend the shelf life of corn oil and protect it from oxidation. The results obtained showed that the addition of EPPE increased the physical parameters of the CH-gel film in terms of T and D. Furthermore, the overall colour characteristics improved from transparency to impenetrability. These films had an additional amount of EPPE in them, which resulted in a substantial (*p* < 0.05) improvement in TS. Increases in EPPE levels, on the other hand, resulted in substantial (*p* < 0.05) decreases in WVP, S, OAR%, and EB%. The SEM analysis confirmed the interactions between EPPE and CH by revealing a consistent structure for all Ch-EPPE films. The EPPE films demonstrated a significant (*p* < 0.05) enhancement in antioxidant and antimicrobial activity. The corn oil PV and TBA values were much lower in the CH-based EPPE gel films throughout the storage experiment. These findings indicate that EPPE films offer an environment-friendly active packaging alternative to synthetic polymers for use in the food industry.

## 4. Materials and Methods

### 4.1. Materials

The empty pea pods were bought from the Kaha Food Canning Company in Kaha, Egypt. They were cleaned and disinfected with sodium hypochlorite at a 50 ppm concentration after they arrived at the lab. They were then cut into small pieces (strips) that were about 2 cm long, placed in a single layer on stainless steel trays, and dried for 12 h at 55 °C in a hot-air oven (Memmert, UF). Then, the dried materials were ground in a FX1000 electrical grinder (Black & Decker, London, England) to pass through a 150 m sieve [25]. Each sample’s dried powder was stored at 4 °C and sealed inside an airtight Kilner jar.

### 4.2. Empty Pea Pods Extract (EPPE)

Following the method outlined by Pinchao-Pinchao et al. [55], ultrasonic-assisted extraction (Elmasonic, Singen, Germany) was used to extract polyphenol components from powdered EPP. The best conditions (30 °C, 20 min, 50% ethanol concentration, and a liquid solid ratio of 1:40) were utilized to prepare EPPE with ethanol as the solvent. The extracted material was wrapped in aluminium foil and kept at −18 °C. Under the influence of a nitrogen gas stream, the solvent was eliminated.

### 4.3. Gel Film Preparation

The CH-gel film-forming solution was made in the manner described by Siripatrawan and Harte [29], with minor modifications. A number of initial experiments were carried out to determine the best type and amount of acid solvent and plasticizer to be utilized in the preparation of CH-based gel films. The findings showed that 2% CH in 1% acetic acid might be used to create the best CH-gel films. The findings also showed that the addition of glycerol as a plasticizer at a percentage of 30% w/w of CH powder enhanced the mechanical characteristics of the films. Hundred millilitres of glacial acetic acid solution (1%) and CH powder (2 g) (deacetylation degree: 75%, Sigma Aldrich Company, Darmstadt, Germany) were combined to create a film-forming solution. The film-forming solution was supplemented with glycerol (El-Gomhoria Chemical Co., Tanta, Egypt), a plasticizer, at a fixed percentage of 30% weight/weight of CH. The resulting solution was then heated in a water bath shaking incubator for 30 min at 60 °C and 100 rpm. To get rid of undissolved contaminants, the chitosan solution was then filtered using a coarse sintered glass filter. After being cooled to ambient temperature, the EPPE was mixed into the film-forming solution to produce percentages of 0, 1, 3, and 5% (*w*/*v*). The resultant solutions were homogenized with a Moulinex homogenizer (Courneuve, France). After that, a sonicator (Singen, Germany) was used to degas the film-forming solutions to get rid of air bubbles. A ceramic plate was used to cast each film-forming solution, and it was allowed to dry there. Prior to testing, the films were conditioned for 48 h at 25 °C and 50% RH in a chamber.

### 4.4. Physical Properties

#### 4.4.1. Film Thickness (T)

A digital Mitutoyo Absolute Micrometer (Tokyo, Japan) was used to measure the T of the film. For every film sample, 10 replications were carried out. The mean values were obtained after the measures were conducted at random locations all over the film sample.

#### 4.4.2. Film Density (D)

The film’s weight and volume were used to calculate the D value of each film. The film’s thickness and area were used to compute its volume.

#### 4.4.3. Film WVP

The WVP was determined using the Zhang et al. [56] technique with the necessary changes. In total, 10 g of anhydrous CaCl_2_ was placed in a glass cup with a diameter of 4 cm, and a film measuring 10 × 10 cm was placed on top, supported by rubber strands. The cups were then piled inside the desiccator and filled with sodium chloride saturation solution (75 RH). For up to seven hours, the cup’s weight was registered every hour. Using linear regression, the slope of each line (K) (g/h) was determined. Equation (1) was used to calculate the WVP values.

(1) $$\mathrm{WVP}=\frac{\left(\mathrm{Film}\;\mathrm{thickness}\right)\times K}{\left(\mathrm{Vapor}\;\mathrm{pressure}\;\mathrm{difference}\right)\times \left(\mathrm{Film}\;\mathrm{area}\right)}$$

#### 4.4.4. Film Water Solubility(S)

The technique of Rambabu et al. [37] was used to determine the film S percentage. The samples were cut into thin strips (20 × 20 mm) and dried in a hot-air oven for 24 h at 105 °C to a consistent weight. Each dried film sample was submerged for 24 h in 70 mL of distilled water. The film samples were then taken out of the solution and allowed to dry again at 100 °C for 24 h. Final weights were noted, and solubility was determined as follows:

(2) $$S\;\left(\%\right)=\frac{\left(\mathrm{Initial}\;\mathrm{dry}\;\mathrm{wieght}\;-\;\mathrm{Final}\;\mathrm{dry}\;\mathrm{wieght}\;\right)}{\mathrm{Initial}\;\mathrm{dry}\;\mathrm{wieght}!}\times 100$$

#### 4.4.5. Films’ ORA

The Wang et al. [49] technique was slightly modified to determine the ORA of the films. In a hot-air oven set at 50 °C, the filter paper (6 cm in diameter) was dried to a consistent weight. The film samples (4 cm × 4 cm) were placed upside down on the filter paper for 48 h while being secured with ropes on the top of glass test tubes containing 5 mL of oil. After 48 h, the filter paper was weighed, and the oil absorption rate (OAR) was determined using the equation

(3) $$\mathrm{OAR}\;\left(\%\right)=\frac{W-W1\;}{W1}\times 100$$

where W is the weight of filter paper after 48 h and W1 is the weight of dried filter paper.

### 4.5. Optical Characteristics

Colour measurement and opacity estimates were used to conduct an optical investigation of the Ch-EPPE films. Using a Miniscan EZ colorimeter (HunterLab), the values of the Hunter colour (L*, a*, and b*) were calculated. Equation (2) was used to calculate the film’s whiteness index using these values [57]. Six readings were taken at various locations on each film. Five film samples were utilized in each replication, which involved five replications for each treatment.

(4) $$\mathrm{Whiteness}\;\mathrm{index}\;=100-\sqrt{a^{2}+\left(100-L\right)2+b^{2}}$$

Utilizing the technique developed by Rambabu et al. [37], the opacity of the film was determined by measuring the absorbance of a rectangular film sample (1 × 4 cm in size) using a UV-Vis spectrophotometer (UV-3600, Shimadzu, Baltimore, MD, USA) with an absorbed wave length of 600 nm. The film’s opacity was calculated using Equation (5).

(5) $$\mathrm{Opacity}=\frac{\mathrm{Absorbance}\;\mathrm{at}\;600\;\mathrm{nm}}{\mathrm{Film}\;\mathrm{thickness}\;\left(\mathrm{mm}\right)}$$

High transparency values, based on this equation, indicate poorer transparency and a greater level of opacity.

### 4.6. Mechanical Properties

The percentages of EB and TS, among other mechanical parameters according to ASTM [58], were assessed using a TA.XT Plus texture analyzer (Stable Micro-Systems, British). A dimension of 1.5 × 10 cm was made from films that had the same thickness. Forced paper was positioned between the two metal grips to support the ends of the film samples. Following machine calibration, the speed was set at 50 mm/min for initial grip separation and 100 mm/min for detection speed. Following the creation of the stress-strain curves, the TS and EB were determined using the following equations:

(6) $$\mathrm{TS}\left(\mathrm{MPa}\right)=\frac{\mathrm{Maximum}\;\mathrm{extension}\;\mathrm{force}\;}{\mathrm{Initial}\;\mathrm{crosssectional}\;\mathrm{area}}\times 100$$

(7) $$\mathrm{EB}\left(\%\right)=\frac{\;\mathrm{Film}\;\mathrm{extension}\;\mathrm{ratio}}{\mathrm{Initial}\;\mathrm{length}}\times 100$$

### 4.7. Bioactivities of CH-EPPE Film

#### 4.7.1. Biodegradation Test

The composting experiment described by Riaz et al. [32] was used to measure the biodegradation capability of the films produced. The topsoil was obtained from Kafrelshiekh University’s experimental field (Kafr El-Shiekh, Egypt). The topsoil was put in a plastic container, and samples of each sheet (2 × 2 cm) were buried at a depth of 2 cm for three weeks. Twice a day, water was sprayed on the soil. The film samples were removed at the end of each week, and the weight loss of each film was recorded.

#### 4.7.2. Antioxidant Properties

##### TPC Measurement

The Folin Ciocalteu reagent was used to estimate the EPPE films’ TPC, as mentioned by Ruiz-Navajas et al. [59]. In order to partially dissolve the films and release the EPPE for the next tests, the film samples (25 mg) were soaked in ethanol (3 mL). The gel film’s total phenolic content was determined.

##### Antioxidant Activity Measurement

The antioxidant activity % of the CH film was measured using the DPPH and ABTS tests outlined by Liu et al. [60]. In brief, 1.5 mL of the film extract was combined with 0.5 mL of a 0.1 mM ethanolic DPPH solution and stored in the dark for half an hour. The DPPH scavenging capacity % was determined from Equation (8) based on the absorbance at 515 nm.

(8) $$\mathrm{DPPH}\;\mathrm{scavenging}\;\left(\%\right)=\left[1-\frac{\;\mathrm{film}\;\mathrm{extract}\;\mathrm{absorbation}}{\mathrm{control}\;\mathrm{absorbation}}\right]\times 100$$

For the test of ABTS scavenging %, the film samples (10 mg) and 3 mL of ABTS stock solutions were combined, absorbance at 734 nm was measured, and scavenging activity was estimated using the following equation:

(9) $$\mathrm{ABTS}\;\mathrm{scavenging}\;\left(\%\right)=\left[1-\frac{\;\mathrm{film}\;\mathrm{extract}\;\mathrm{absorbation}}{\mathrm{control}\;\mathrm{absorbation}}\right]\times 100$$

##### Migration Test

The migration assessment was carried out in accordance with the method of Oliveira et al. [61]. CH-gel film specimens were chopped into small (2 × 2 cm) pieces and soaked in 5 mL water (to imitate an aqueous medium) and 95% ethanol (to simulate a fatty medium). Seven days were spent shaking it at 125 rpm at 25 °C. The TPC approach described in Section 4.7.2 was used to determine the migration of active molecules into food simulants.

##### Antimicrobial Activity

According to the method outlined by Elsebaie and Essa [62], the antimicrobial activity of the prepared gel films was assessed against two Gram-positive, *Bacillus subtilis* ATCC21331 and *Pseudomonas aeruginosa* (CGMCC1.8721), and two Gram-negative, *Escherichia coli* ATCC25921 and *Salmonella typhimurium* (CGMCC 1.10754), bacteria. A movable calliper was used to measure the inhibitory zone.

### 4.8. SEM Scanning

A Carl Zeiss SEM (EVO-LS-10, Hamburg, Germany) was used to examine the films created at 10 kV. Film sections with dimensions of 1 × 1 cm were cut out, fastened on aluminium stubs with carbon tape, and coated with gold on the outside. Additionally, the image was adjusted to ×5000.

### 4.9. Film Application in Corn Oil Packaging

The film specimens were stored at 50 °C for 10 days after being wrapped in line rope and placed on top of glass bottles containing corn oil. On days 0, 2, 4, 6, 8, and 10, a sample of 5 mL was removed from each glass tube in order to calculate the PV and TBA values using the technique outlined by Elsebaie et al. [63]. The analysis was carried out three times, and the average values were provided. A glass container with no film was applied as a control.

### 4.10. Statistical Analysis

To analyze differences between values, an ANOVA was performed using SPSS (Ver. 16.0, 2007)’s general linear regression model. For statistical testing, the probability degrees of *p* < 0.05 were substantially considered. Triplicates for each measurement and experiment were carried out.

## Figures and Tables

**Table 1:** Thickness (T), density (D), water vapour permeability (WVP), solubility in water (S), and oil absorption ratio (OAR %) of the chitosan films modified with different percentages of EPPE.

Film Samples	Film Properties				
	T (mm)	D (g/cm^3^)	WVP (×10^−10^ g^−1^ s^−1^ pa^−1^)	S (%)	ORA (%)
**Control** **(Ch-0% EPPE)**	0.132 ± 0.08 ^d^	1.13 ± 0.02 ^d^	2.34 ± 0.04 ^a^	29.40 ± 1.23 ^a^	0.31 ± 0.006 ^a^
**Ch-1% EPPE**	0.167 ± 0.09 ^c^	1.52 ± 0.03 ^c^	1.58 ± 0.09 ^b^	28.17 ± 1.78 ^a^	0.26 ± 0.004 ^b^
**Ch-3% EPPE**	0.189 ± 0.06 ^b^	1.75 ± 0.02 ^b^	1.32 ± 0.07 ^b^	22.43 ± 2.11 ^b^	0.15 ± 0.003 ^c^
**Ch-5% EPPE**	0.216 ± 0.08 ^a^	1.94 ± 0.02 ^a^	1.08 ± 0.06 ^c^	18.75 ± 1.94 ^c^	0.08 ± 0.001 ^d^

Data are presented as mean ± SD. Means with different superscripts (^a–d^) in lowercase letters in a column are significantly different at *p* < 0.05.

**Table 2:** Colour parameters and optical index of chitosan films modified with different percentages of EPPE.

Film Samples	L*	b*	a*	Whiteness Index	Opacity
**Control** **(Ch-0% EPPE)**	88.17 ± 0.55 ^a^	1.03 ± 0.01 ^a^	0.84 ± 0.01 ^a^	88.10 ± 0.43 ^a^	0.71 ± 0.02 ^a^
**Ch-1% EPPE**	85.40 ± 0.72 ^b^	1.19 ± 0.07 ^b^	0.87 ± 0.01 ^a^	85.33 ± 0.51 ^b^	0.80 ± 0.03 ^b^
**Ch-3% EPPE**	80.35 ± 0.61 ^c^	1.47 ± 0.05 ^c^	0.94 ± 0.03 ^a^	80.27 ± 0.60 ^c^	0.97 ± 0.03 ^c^
**Ch-5% EPPE**	77.64 ± 0.66 ^d^	1.98 ± 0.04 ^d^	1.12 ± 0.03 ^b^	77.53 ± 0.48 ^d^	1.23 ± 0.04 ^d^

Data are presented as mean ± SD. Means with different superscripts (^a–d^) in lowercase letters in a column are significantly different at *p* < 0.05.

**Table 3:** Mechanical properties of chitosan films modified with different percentages of EPPE.

Film Samples	Tensile Strength (MPa)	Elongation at Break (EB) %
**Control** **(Ch-0% EPPE)**	21.30 ± 1.19 ^d^	53.42 ± 3.02 ^d^
**Ch-1% EPPE**	23.16 ± 1.23 ^c^	54.17 ± 2.98 ^c^
**Ch-3% EPPE**	25.92 ± 1.40 ^b^	56.83 ± 2.77 ^b^
**Ch-5% EPPE**	26.87 ± 1.38 ^a^	58.64 ± 3.00 ^a^

Data are presented as mean ± SD. Means with different superscripts (^a–d^) in lowercase letters in a column are significantly different at *p* < 0.05.

**Table 4:** Migration test of chitosan films modified with 0 to 5% EPPE.

Film Samples	Simulant Type	
	Total Phenolic Content (mg gallic acid/mL Water)	Total Phenolic Content (mg gallic acid/mL Ethanol)
**Control** **(Ch-0% EPPE)**	0.002 ± 0.000 ^a^	0.004 ± 0.001 ^a^
**Ch-1% EPPE**	1.04 ± 0.05 ^c^	1.92 ± 0.09 ^c^
**Ch-3% EPPE**	3.19 ± 0.03 ^b^	5.06 ± 0.06 ^b^
**Ch-5% EPPE**	5.09 ± 0.06 ^a^	9.12 ± 0.08 ^a^

Data are presented as mean ± SD. Means with different superscripts (a–d) in lowercase letters in a column are significantly different at *p* < 0.05.

**Table 5:** Antibacterial activity of chitosan films modified with different percentages of EPPE.

Film Samples	Inhibition Zone Diameter (mm)			
	*Salmonella typhimurium*	*E. coli*	*Bacillus subtilis*	*Pseudomonas aeruginosa*
**Control** **(Ch-0% EPPE)**	NA	NA	NA	NA
**Ch-1% EPPE**	7.89 ± 0.10 ^c^	8.12 ± 0.15 ^c^	10.67 ± 0.13 ^c^	10.35 ± 0.16 ^c^
**Ch-3% EPPE**	11.38 ± 0.17 ^b^	12.63 ± 0.12 ^b^	15.94 ± 0.14 ^b^	15.41 ± 0.11 ^b^
**Ch-5% EPPE**	15.66 ± 0.14 ^a^	16.25 ± 0.10 ^a^	19.42 ± 0.20 ^a^	18.98 ± 0.18 ^a^

Data are presented as mean ± SD. Means with different superscripts (a–d) in lowercase letters in a column are significantly different at *p* < 0.05. NA means non-active.

## Data Availability

The authors confirm that the data supporting the findings of this study are available within the article.
